# Antibody Profile Comparison against MSP1 Antigens of Multiple *Plasmodium* Species in Human Serum Samples from Two Different Brazilian Populations Using a Multiplex Serological Assay

**DOI:** 10.3390/pathogens10091138

**Published:** 2021-09-04

**Authors:** Eliana Ferreira Monteiro, Carmen Fernandez-Becerra, Izilda Curado, Gerhard Wunderlich, Meire Ioshie Hiyane, Karin Kirchgatter

**Affiliations:** 1 Instituto de Medicina Tropical, Faculdade de Medicina, Universidade de São Paulo, São Paulo 05403-000, Brazil; elianafmonteiro@usp.br (E.F.M.); izcurado@gmail.com (I.C.); 2 ISGlobal, Hospital Clínic—Universitat de Barcelona, 08036 Barcelona, Spain; carmen.fernandez@isglobal.org; 3 Germans Trias i Pujol Health Science Research Institute (IGTP), 08916 Badalona, Spain; 4 Departamento de Laboratórios Especializados, Superintendência de Controle de Endemias, São Paulo 01027-000, Brazil; 5 Departamento de Parasitologia, Instituto de Ciências Biomédicas, Universidade de São Paulo, São Paulo 05508-000, Brazil; gwunder@usp.br; 6 Departamento de Imunologia, Instituto de Ciências Biomédicas, Universidade de São Paulo, São Paulo 05508-900, Brazil; miy@icb.usp.br

**Keywords:** malaria, *Plasmodium malariae*, MSP1, serology, Brazil, multiplex bead assay

## Abstract

*Plasmodium malariae* has a wide geographic distribution, but mainly at very low parasitemias and in co-infections, leading to an underestimated prevalence of this species. Studies for the detection of antibodies against *Plasmodium* recombinant proteins are increasingly used to map geographical distributions, seroprevalence and transmission intensities of malaria infection. However, no seroepidemiological survey using recombinant *P. malariae* proteins has been conducted in Brazil. This work evaluated the antibody response in serum samples of individuals from endemic regions of Brazil (the Amazon region and Atlantic Forest) against five recombinant proteins of *P. malariae* merozoite surface protein 1 (MSP1), and the MSP1 C-terminal portions of *P. vivax* and *P. falciparum*, in a multiplex assay. The positivity was 69.5% of samples recognizing at least one MSP1 recombinant protein. The mean of the Reactivity Index for the C-terminal portion of the *P. falciparum* was significantly higher compared to the other recombinant proteins, followed by the C-terminal of *P. vivax* and the N-terminal of *P. malariae*. Among the recombinant *P. malariae* proteins, the N-terminal of *P. malariae* showed the highest Reactivity Index alone. This study validates the use of the multiplex assay to measure naturally acquired IgG antibodies against *Plasmodium* MSP1 proteins and demonstrate that these proteins are important tools for seroepidemiological surveys and could be used in malaria surveillance.

## 1. Introduction

*Plasmodium malariae* is one of the oldest parasites that cause malaria in humans but is also one of the most neglected, probably due to their lower prevalence and disease severity, as compared to other human *Plasmodium* spp. [1]. However, *P. malariae* is widespread throughout sub-Saharan Africa, Southeast Asia, Indonesia, South America and on islands of the western Pacific [2]. Recently, a systematic review and meta-analysis study investigated the global trend of *P. malariae* infections in the last two decades, showing an overall seroprevalence of 2%, with the highest prevalence (3.16%) in the African region and the lowest prevalence (0.06%) in the Eastern Mediterranean region, whereas the American region had the second highest *P. malariae* prevalence (2.94%) [3]. 

*Plasmodium malariae* also occurs more likely in co-infections than mono-infections [3,4]. However, it is known that, in cases of mixed infections, the presence of *P. malariae* is rarely apparent, and low-level or subpatent infections are revealed only by molecular techniques [2]. Moreover, it has been suggested that the prevalence and distribution of *P. malariae* may be underestimated due to its misrecognition in thick blood smears, often leading to a mistaken identification as *P. vivax* [5].

Despite the minor number of cases reported in many parts of the world, a significant part of the population shows serologic evidence of recent exposure to *P. malariae*, suggesting that asymptomatic infections with this species may be frequent [6,7,8,9]. In fact, *P. malariae* is known to produce low parasitemia infections that are frequently non-symptomatic and long-lasting [10], possibly due its reduced growth rate [11] and preference for older erythrocytes [12].

In a general way, subclinical malaria arises due to naturally acquired immunity, directly related to both the age and the many years of exposure of the individual to the malaria parasites [13,14]. Asymptomatic infections can play a central role in the maintenance of diseases, making them difficult to control. The World Health Organization (WHO) has several strategic programs to eliminate malaria. These are based mainly on the identification and treatment of symptomatic cases, and with a particular focus on *P. falciparum* and *P. vivax* malaria [15]. Their decline could provide a favorable ecological niche for other malaria parasites, such as *P. malariae* [16]. Thus, in order to contribute to the elimination of malaria in endemic countries, mass diagnosis and treatment aiming to remove this residual transmission source is necessary. 

Serological assays can measure past exposure identifying at-risk populations and help to produce risk maps to improve the targeting of interventions [17]. Therefore, we evaluated the presence of antibodies against *Plasmodium* merozoite surface protein 1 (MSP1) in human serum samples using a multiplex serological assay [18]. MSP1 is a cell surface protein involved in the erythrocyte invasion process. It is the most abundant protein in the malaria parasite [19] and has been demonstrated to be an important tool for seroepidemiological surveys of malaria [20]. In Brazil, malaria caused by *P. vivax*, *P. falciparum* and *P. malariae* occurs predominantly in the Amazon region, where it is a serious public health problem [21]. In the Atlantic Forest, autochthonous cases of malaria caused by *P. vivax* and *P. malariae* are transmitted and maintained in small foci of transmission with very specific characteristics [21]. A multiplex serological method was used, with MSP1 recombinant proteins of *P. malariae* (PmMSP1_F1_, PmMSP1_F2_, PmMSP1_F3_, PmMSP1_F4_ and PmMSP1_19_), *P. falciparum* (PfMSP1_19_) and *P. vivax* (PvMSP1_19_), and the sera of individuals from Acre and Rondônia, in the Amazon region, and Intervales, in the Atlantic Forest, to establish the prevalence of antibodies against these parasite species in different endemic areas.

## 2. Results

### 2.1. Analysis of Coupling Efficiency of Glutathione S-Transferase (GST)-Fusion Proteins to Bio-Plex Carboxylated Magnetic Beads

**Figure A1:**
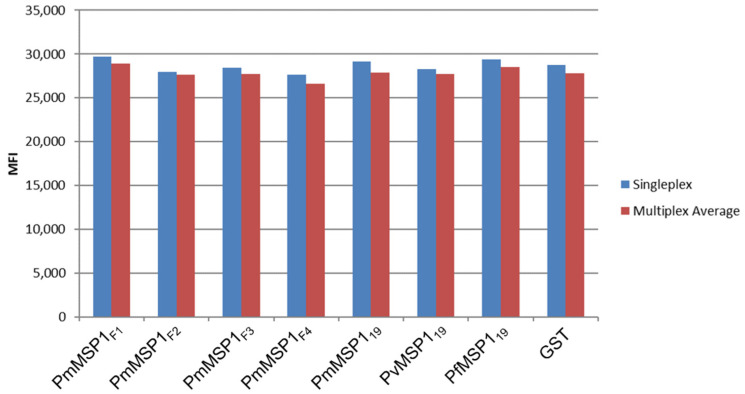
Coupling efficiency of the recombinant proteins. Comparison of the median fluorescence intensity (MFI) values between the singleplex and multiplex assays. The recombinant proteins present on the surface of the beads were detected using biotinylated anti-GST antibody and streptavidin-R-phycoerythrin. The reading was performed on the Bio-Plex 200 (Bio-Rad) instrument as described in “Methods”.

The efficiency of the coupling of each protein was determined by the analysis in singleplex, and later evaluated in a multiplex bead assay (MBA), where it played the role of quality control in all of the sample plates tested. For this, beads were covalently coated with the different recombinant proteins and tested with a biotinylated anti-GST antibody. The graph in Appendix A (Figure A1) shows that the fluorescence, presented as median fluorescence intensity (MFI), remained stable in both reactions, with minimal variation between the assays and very similar MFI values for all of the proteins.

### 2.2. Cut-Off Determinations

**Figure A2:**
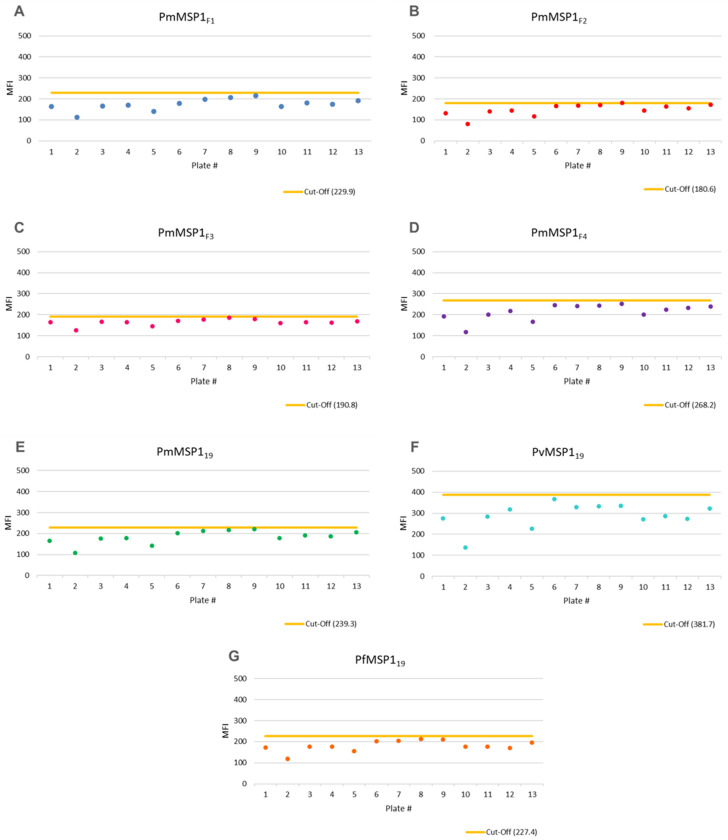
Distribution of the MFI values obtained for the recombinant MSP1 proteins from *P. malariae* (**A**–**E**), *P. vivax* (**F**) and *P. falciparum* (**G**). The MFI values represent the average value of the duplicates of the negative controls (un-infected individuals) present on each assay plate. The horizontal line represents the cut-off of each recombinant protein calculated as the geometric mean plus three standard deviations.

To determine the cut-off values for each recombinant protein in each plate, sera from uninfected individuals were used in duplicate to obtain the MFI values. The cut-off values are presented as the geometric mean of the values, obtained with a panel of eight negative control sera, plus three standard deviations. The averages of the cut-off values are shown in Figure A2. For all of the recombinant proteins, the cut-offs were around 200 MFI, except for PvMSP1_19_ (~380 MFI).

### 2.3. Naturally Acquired Antibody Responses

**Figure 1:**
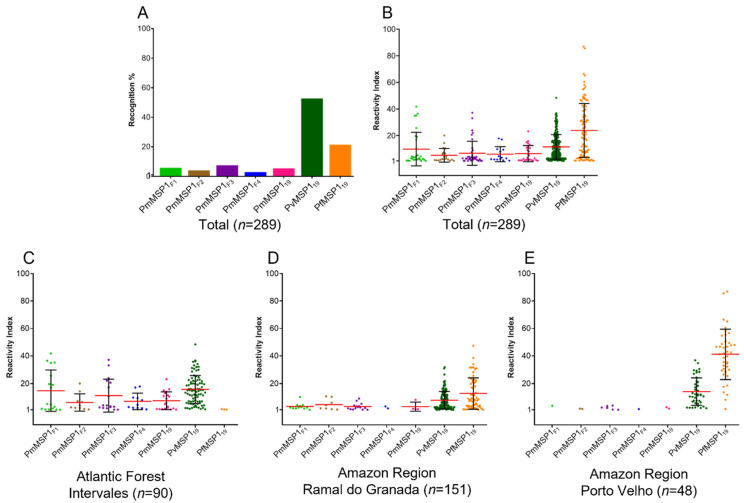
Distribution of the positive serum samples from Brazil according to the recognition of the recombinant *Plasmodium* MSP1 protein (**A**); Reactivity Index (RI) of IgG antibodies against different recombinant MSP1 proteins in sera from individuals exposed to malaria (**B**); RI of IgG antibodies in sera from individuals from Intervales (**C**), Ramal do Granada (**D**) and Porto Velho (**E**). Serum samples were tested in duplicate in multiplex bead assay (MBA) at a 1:100 dilution against seven recombinant proteins. The black bars represent the mean RI (red lines) ± standard deviations obtained for each recombinant protein.

A total of 416 samples from individuals living in two Brazilian endemic regions, the Amazon region (Ramal do Granada and Porto Velho) and the Atlantic Forest (Intervales State Park), were analyzed against seven recombinant proteins (PmMSP1_F1_, PmMSP1_F2_, PmMSP1_F3_, PmMSP1_F4_, PmMSP1_19_, PfMSP1_19_ and PvMSP1_19_). The overall seroprevalence of IgG antibodies against at least one of the recombinant proteins was 69.5% (289 samples). Sera reactive to *P. vivax* PvMSP1_19_ were the most frequent (52.8%), followed by those positive to *P. falciparum* PfMSP1_19_ (21.5%), whereas the percentages of positivity were very low for the five recombinant proteins of *P. malariae* (Figure 1A).

The serological results were normalized by calculating the Reactivity Index (RI), which denotes the ratio between the MFI of the samples and the cut-off values. In a general analysis with sera from all of the three locations together, the RI value was significantly higher for the C-terminal portion of the *P. falciparum* recombinant protein (PfMSP1_19_) compared to the other recombinant proteins (*p* < 0.0001, ANOVA), followed by the C-terminal of *P. vivax* (PvMSP1_19_) and the N-terminal of *P. malariae* (PmMSP1_F1_), the latter being the most reactive among all *P. malariae* recombinant proteins tested. However, the RI value for the PmMSP1_F1_ was non-significantly higher (*p* > 0.8529, ANOVA) when compared to the other *P. malariae* recombinant proteins (Figure 1B).

The analysis of individual values of the RI of IgG antibodies in sera from individuals from Intervales, Ramal do Granada and Porto Velho by locality showed that individuals seropositive for *P. vivax* had a high RI in all of the three locations (means 15.73, 7.86 and 14.12, respectively) (Figure 1C–E). However, in the localities from the Amazon region, the recognition for PfMSP1_19_ was even greater (means 12.80 and 41.18 for Ramal do Granada and Porto Velho, respectively) (Figure 1D–E). 

In the Atlantic Forest region, sera reactivity against the recombinant proteins PmMSP1_F1_ and PmMSP1_F3_ stood out with high RIs (41.7 and 37.2, respectively), and three samples were found that recognized PfMSP1_19_ with an RI close to 1.0 (Figure 1C). In Ramal do Granada, the RI values were low for *P. malariae*, and high for *P. falciparum* (Figure 1D). In Porto Velho, *P. malariae* had the lowest indexes and *P. falciparum* had the highest RI mean of all analyses (Figure 1E), with a significant difference in relation to Ramal do Granada (*p* < 0.0001).

**Figure 2:**
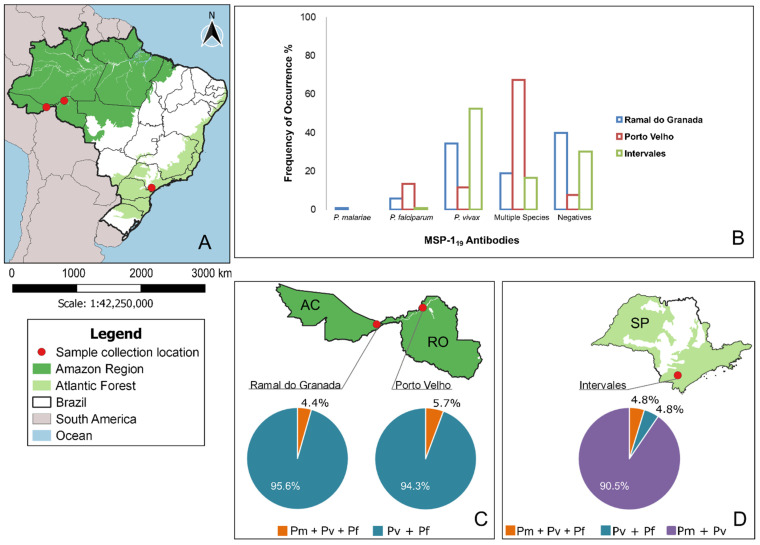
Naturally acquired antibody responses to recombinant PmMSP1_19_, PvMSP1_19_ and PfMSP1_19_ antigens in three localities on the map of Brazil (**A**). Antibody prevalences to each of the MSP1 recombinant proteins are shown for individuals from the three localities in Brazil (**B**). Distribution of antibodies against multiple *Plasmodium* species are shown for Amazon region (**C**) and Atlantic Forest (**D**).

In the comparison of seroprevalence between samples from the Amazon region and the Atlantic Forest with their diverging malaria environment (Figure 2A), only the three MSP1_19_-specific antibodies (*P. vivax*, *P. falciparum* and *P. malariae*) were evaluated, in order to prevent bias towards *P. malariae*. Thus, Figure 2 shows the *Plasmodium* MSP1_19_ antibody prevalence in serum samples from Brazil according to the recognition for the three recombinant proteins in each locality analyzed (PmMSP1_19,_ PfMSP1_19_ and PvMSP1_19_) (Figure 2B). Data about isolated recognition showed high positivity rates for PvMSP1_19_ in Ramal do Granada and Intervales, whereas the recognition of PfMSP1_19_ was more frequent than PvMSP1_19_ in Porto Velho (Figure 2B). The prevalence of antibodies against more than one *Plasmodium* species was higher than single-species antibody prevalence in Porto Velho (Figure 2B). The single-species *P. malariae* seroprevalence was rare, being found only in Ramal do Granada (Figure 2B). The overall multiple-species antibody presence exhibited an important difference in the distribution of the combinations within the two regions, showing *P. vivax* and *P. falciparum* in the Amazon region (Figure 2C) and *P. vivax* and *P. malariae* in the Atlantic Forest (Figure 2D). 

**Figure 3:**
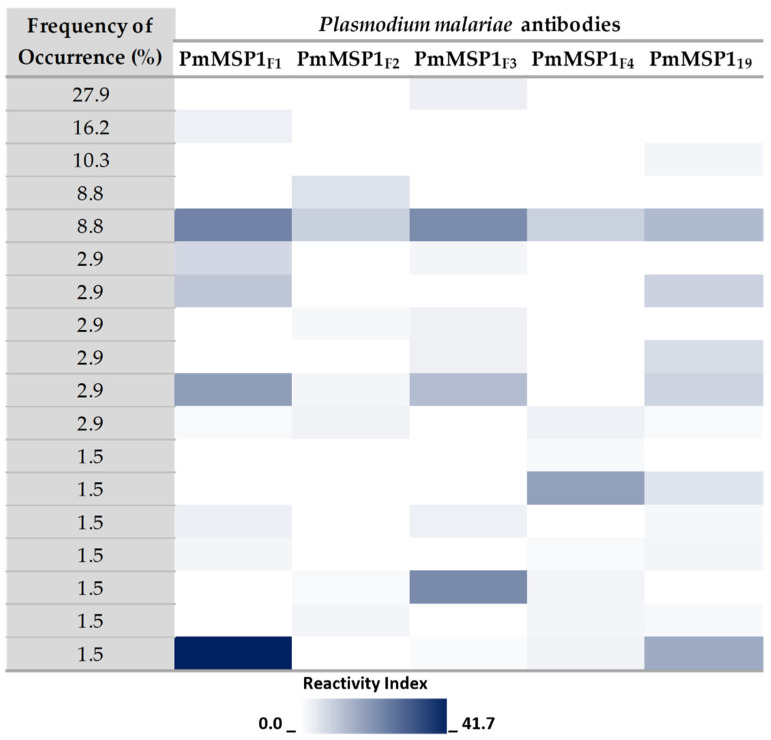
Heatmap of antibody levels against each PmMSP1 recombinant protein, considering only PmMSP1 positive samples. Each horizontal line represents a combination and the depicted colored blocks in the line correspond to the Reactivity Index (RI) to each protein of *P. malariae*. RI values increase with more intense blue color.

In the analysis of the recognition of the five *P. malariae* proteins, PmMSP1_F1_, PmMSP1_F2_, PmMSP1_F3_, PmMSP1_F4_ and PmMSP1_19_ were recognized by 41.2%, 29.4%, 52.9%, 20.6% and 38.2% of the *P. malariae* positive sera (*n* = 68), respectively. A comparison was performed by the eighteen distinct combination patterns obtained, the frequencies of which are shown in Figure 3. PmMSP1_F3_ was recognized distinctly with a frequency of 27.9%, whereas 16.2% of the sera were solely positive for PmMSP1_F1_. The five *P. malariae* recombinant proteins together (PmMSP1_F1_, PmMSP1_F2_, PmMSP1_F3_, PmMSP1_F4_ and PmMSP1_19_) were recognized in 8.8% of the *P. malariae*-positive samples, showing RI values considered high in our sample set scale (Figure 3). A total of 1.5% of the samples presented antibodies against the N-terminal portion (PmMSP1_F1_) and also the C-terminal portion (PmMSP1_19_), with the highest RI values obtained for these proteins (41.7 and 15.9, respectively) (Figure 3).

## 3. Discussion

Studies for the detection of antibodies against *Plasmodium* MSP1 proteins are increasingly used to map geographical distributions, seroprevalence and transmission intensities of malaria infection [22,23,24]. The C-terminal portion of the merozoite surface protein 1 (MSP1_19_) is a promising candidate for the malaria vaccine, as it is highly immunogenic [25,26], therefore being extensively analyzed as an immunological target in many studies [23,25,26,27,28]. On the other hand, many studies also use the N-terminal portion of MSP1, which elicits a strong IgG response in *P. vivax* and *P. falciparum* infections [29,30,31,32]. However, although there are already some studies using *P. malariae* MSP1 [8,33], no seroepidemiological survey has been carried out in Brazil, leaving gaps regarding the parasite’s occurrence in different malaria endemic settings in Brazil. Previous studies performed by our group have already shown that recombinant PmMSP1 proteins can be useful diagnostic markers of *P. malariae* in epidemiological studies [34]. Here, we analyzed the prevalence of individuals in endemic areas of malaria in Brazil with naturally acquired antibodies against seven recombinant proteins, including *P. malariae* (PmMSP1_F1_, PmMSP1_F2_, PmMSP1_F3_, PmMSP1_F4_ and PmMSP1_19_), *P. falciparum* (PfMSP1_19_) and *P. vivax* (PvMSP1_19_).

Antibodies against PvMSP1_19_ were identified in more than half of our sample set, followed by those for PfMSP1_19_, and in smaller percentages by those against the different recombinant proteins of *P. malariae* (PmMSP1_F1_, PmMSP1_F2_, PmMSP1_F3_, PmMSP1_F4_ and PmMSP1_19_), corroborating with the data obtained in Brazilian historical data of malaria that have demonstrated the predominance of *P. vivax* over *P. falciparum* over the years [35,36]. However, comparing the recognition of the recombinant proteins using the RI obtained for each protein, very similar levels were obtained for PvMSP1_19_ and *P. malariae*, mainly for PmMSP1_F1_ and PmMSP1_F3_ in the Atlantic Forest region, a documented area for the presence of *P. malariae* infections [37]. 

The highest Reactivity Index for IgG antibodies among the three species was found for *P. falciparum* mainly in Porto Velho, a known hotspot for this species in Brazil [35]. The C-terminal of the *P. falciparum* MSP1 protein, PfMSP1_19_, has already shown to be related to the acquisition of clinical immunity, which is probably cumulative, with the expansion and refinement of a repertoire of antibodies, protecting the individual from severe malaria, which is a rare event in the Amazon region [38].

In the Atlantic Forest, although only a small number of clinical cases of malaria are registered, a large portion of the population shows serological evidence of recent exposure to *P. vivax* or *P. malariae* [6,39], suggesting a high prevalence of asymptomatic infections that can act as reservoirs of infection, sustaining the transmission and undermining malaria eradication and control strategies [13,40,41]. In our study, individuals residing in the Atlantic Forest area showed high rates of reactivity of IgG antibodies against the recombinant proteins of *P. malariae* (PmMSP1_F1_, PmMSP1_F2_, PmMSP1_F3_, PmMSP1_F4_ and PmMSP1_19_,) compared to other locations, reflecting a cumulative transmission profile. This suggests that these individuals may be frequently exposed to infections caused by this species or even maintain these infections for long periods, since *P. vivax* infections induce long-lasting memory B-cell responses even in settings with very low transmission [42]. 

Interestingly, three samples from the Atlantic Forest recognized the C-terminal of *P. falciparum* (PfMSP1_19_) with very low RI, in concordance with previous reports [39,43,44]. Infection by *P. falciparum* in the Atlantic Forest has been previously detected by PCR in individuals from Intervales, who did not present classical symptoms and reported not having travelled to malaria-endemic areas, such as the Amazon region [39]. A cross-sectional study carried out on humans living on the border of the Atlantic tropical rainforest region of Rio de Janeiro identified *P. falciparum* in humans with malaria [45]. Recent evidence points to the presence of *P. falciparum* in a silent cycle, detected only by molecular methods in asymptomatic individuals [37].

The overall seroprevalence of the highly immunogenic PvMSP1_19_ in the Brazilian Amazon was in line with other reports from this endemic area, where nearly 70% of the studied population carried PvMSP1_19_ antibodies [46]. Similarly, *P. falciparum* also showed a high RI of IgG antibodies in the Amazon region, showing a profile of constant exposure to parasites, as demonstrated in previous studies [38,47].

It is important to note that 60% of our study participants with positive results harbored single-species antibodies against MSP1_19_, endorsing the idea that IgG antibody responses to the utilized malaria MSP1 antigens appear to be species-specific [33].

The possible combinations of antibodies were analyzed in order to obtain information on the combined occurrence of specific antibodies against *P. malariae*. Interestingly, the recognition of the five fragments combined was formed by samples with average Reactivity Indexes. However, PmMSP1F1 and PmMSP1F3 were recognized singly in a high frequency, showing the need to use the various domains of PmMSP1 in combination during serological surveys focusing on this species. Analyzing the eighteen recognition patterns that were obtained, the PmMSP1_F1_ protein is present in 50% of them, which may indicate the potentially high immunogenicity of this MSP1 region of *P. malariae*, as has already been demonstrated for *P. vivax* and *P. falciparum* MSP1 [30,31,32,41]. 

In the Amazon region, *P. malariae* cases are rarely identified [35]. However, our results indicate a prevalence of this parasite in 5% of the mixed infections occurring in the Amazon. With the current predominance of *P. vivax* and still a certain prevalence of *P. falciparum*, combined with low parasitaemia and all of the difficulties in diagnosing *P. malariae*, it is not surprising that *P. malariae* infections have been overlooked in this area. Though not as clinically relevant, it is becoming more appreciated that donors with asymptomatic *P. malariae* infection could be reservoirs of transfusion-transmitted malaria [48], and serological surveillance through the MBA offers a prime opportunity for a robust identification of regions where this parasite may be endemic. Alternatively, these PmMSP1 recombinant proteins could be useful to the species-specific diagnosis of *P. malariae* in routine malaria diagnosis by rapid diagnostic tests (RDTs), which currently target *P. falciparum*, and other *Plasmodium* species are identified as “pan-species” [49]. 

## 4. Materials and Methods

### 4.1. Collection of Serum Samples

All of the samples used in this study were collected during research projects that have been published previously [6,36,38] and were deposited in a biorepository at the Institute of Biomedical Sciences of the University of São Paulo (registered by CEPSH 020/2015). Their use in this study was approved by the Ethics Committee on Human Research (CEPSH number 100,3485 of 04/15/2015). A total of 416 serum/plasma samples were obtained from individuals from two malaria endemic regions in Brazil: the Amazon and Atlantic Forest regions. 

Sera from the Amazon region were received from two different localities in the Western Brazilian Amazon: (i) 238 samples from an ongoing population-based cohort study accomplished in an agricultural settlement (Ramal do Granada, Acrelândia, Acre state), collected from March 2004 to May 2005 [36]; (ii) 52 samples from a population situated on the riverbanks of the Madeira River, a riverside area of Porto Velho, the capital of Rondônia state, collected from 2006 to 2008 [38]. As in other endemic settings in Brazil, the majority of malaria cases in these areas are caused by *P. vivax* [35].

Sera from the Atlantic Forest were collected in Intervales State Park (126 samples), district of Guapiara, São Paulo state, in January 2002 [39]. This Vale do Ribeira area and coastal areas of the state of São Paulo have reported prevalence of *P. vivax* malaria and few, but not insignificant, infections by *P. malariae* [35].

### 4.2. Recombinant Antigens 

GST-fusion proteins of *P. malariae*, representing the polymorphic N-terminal (PmMSP1_F1_), the central (PmMSP1_F2_, PmMSP1_F3_ and PmMSP1_F4_) and the conserved C-terminal (PmMSP1_19_) regions, were used in parallel with the GST protein alone (as a control), for detecting anti-MSP1 antibodies in the human serum samples. Moreover, C-terminal of *P. vivax* (PvMSP1_19_) and C-terminal of *P. falciparum* (PfMSP1_19_) previously produced [34], were also used. 

### 4.3. Coupling Efficiency Assessment

The overall efficiency of *Plasmodium* spp. (GST-MSP1) to Bio-Plex carboxylated beads in MBAs were performed using a rabbit anti-GST (Biotin) polyclonal IgG antibody (cod. ab87834, Abcam, Cambridge, MA, USA) to detect bead-coupled fusion protein.

Anti-GST antibody was used 1:1000 in the assay buffer (PBS 1×, 1% BSA, Tween 20 0.02%) (50 µL/well). Bound anti-GST antibody was detected with Streptavidin-R-Phycoerythrin (2 µg/mL) (cod. 42250, Sigma-Aldrich, St. Louis, MO, USA) and fluorescence was measured on the Bio-Plex 200 instrument (Bio-Rad, Hercules, CA, USA), as described below. The system includes two lasers: the classification laser (635 nm excitation) for identifying the bead signatures, and the reporter laser (532 nm excitation) for detecting the target.

### 4.4. Recombinant Protein Multiplex Bead Assay (MBA)

The recombinant proteins were covalently linked to Bio-Plex Pro Magnetic COOH Beads using the Bio-Plex Amine Coupling Kit (Bio-Plex Amine Coupling Kit, Bio-Rad, Hercules, CA, USA) following the manufacturer’s instructions. The coupled beads were then used for the analysis of the samples of individuals as described [18], with modifications. Briefly, 50 µL of bead suspension, corresponding to 2000 coated beads, was used with each serum sample. Serum samples were diluted 1:50 in an assay buffer (1 × PBS, 1% BSA, Tween 20 0.02%) and 50 µL aliquots were added to 50 µL protein-coated magnetic beads (final dilution 1:100). Aliquots of 50 µL goat anti-human IgG (γ-chain specific)-Biotin antibody (cod. B1140, Sigma-Aldrich, St. Louis, MO, USA) (diluted 1:2000) and Streptavidin-R-Phycoerythrin (2 µg/mL) (cod. 42250, Sigma-Aldrich, St. Louis, MO, USA) were used in subsequent incubations. The beads were resuspended in 125 µl of assay buffer (1 × PBS, 1% BSA, Tween 20 0.02%) and the fluorescence was measured with the Bio-Plex200 system (Bio-Rad, Hercules, CA, USA). Results were expressed as median fluorescence intensity (MFI).

Sera were tested in two replicates and assessed by the MFI values of antibodies binding to the recombinant proteins, minus the MFI value of the same serum for GST alone. Cut-off values are presented as the geometric mean of values, obtained with a panel of eight negative control sera, plus 3 × standard deviations. Mean cut-off values are shown in Figure A2.

Quantitative results were obtained by normalizing the data using the Reactivity Index (RI). The RI was established using the fluorescence values expressed as MFI for each sample and divided by the cut-off of each plate. Samples with an RI ≥ 1 were considered positive.

## 5. Conclusions

The current study provided the first malaria seroprevalence data for the three circulating *Plasmodium* species (*P. vivax*, *P. falciparum* and *P. malariae*) in two Brazilian areas: in the Amazon region, where malaria is a serious public health problem, and in regions that record a low but persistent number of autochthonous cases, as is the case for the Atlantic Forest, in the extra-Amazonian region. The high rates of IgG antibody reactivity against *P. vivax* recombinant proteins in all localities, *P. malariae* in the Atlantic Forest region and *P. falciparum* in the Amazon region, support the hypothesis that there is a frequent circulation of the parasites in asymptomatic infections that can play the role of potential reservoirs.

Our results also validate the use of the MBA to measure naturally acquired IgG antibodies against *P. malariae* recombinant MSP1 proteins in humans and demonstrate that these proteins are important tools for seroepidemiological surveys that must be considered in malaria surveillance and elimination programs.

## 6. Limitations

The incidence of malaria in Brazil has dropped in the last decade; however, 99% of all malaria cases registered in the country are still stemming from the Amazon region, which is largely due to autochthonous *P. vivax* and *P. falciparum* transmission. Current *P. falciparum* transmission is basically restricted to the same hotspot areas as in 2000 to 2010, when sera were obtained for this study. Similarly, malaria reported in the Atlantic Forest comprises autochthonous *P. vivax* and *P. malariae* transmission without significant modifications in its epidemiology during this period mentioned above [35]. Another limitation of this study is that only different MSP1 proteins from *P. malariae* were used. Other antigenic surface proteins from the parasite, such as the Circumsporozoite protein (CSP), thrombospondin-related anonymous protein (TRAP), Duffy-binding protein (DBP) and apical membrane antigen 1 (AMA1), may also produce valuable results.

## Data Availability

The data presented in this study are openly available in FigShare at [dx.doi.org/10.6084/m9.figshare.16569378].
